# Inter-Person Differences in Isometric Coactivations of Triceps Surae and Tibialis Anterior Decrease in Young, but Not in Older Adults After 14 Days of Bed Rest

**DOI:** 10.3389/fphys.2021.809243

**Published:** 2022-01-28

**Authors:** Matjaž Divjak, Gašper Sedej, Nina Murks, Mitja Gerževič, Uros Marusic, Rado Pišot, Boštjan Šimunič, Aleš Holobar

**Affiliations:** ^1^System Software Laboratory, Institute of Computer Science, Faculty of Electrical Engineering and Computer Science, University of Maribor, Maribor, Slovenia; ^2^Science and Research Centre Koper, Institute for Kinesiology Research, Koper, Slovenia; ^3^Department of Health Sciences, Alma Mater Europaea – ECM, Maribor, Slovenia

**Keywords:** high density electromyography, muscle disuse, motor units, discharge rate, aging

## Abstract

We examined activation patterns of the gastrocnemius medialis (GM), gastrocnemius lateralis (GL), soleus (SO), and tibialis anterior (TA) muscles in eight older (58.4 ± 3.3 years) and seven young (23.1 ± 2.9 years) participants, before and after 14 days of horizontal bed rest. Visual feedback on the exerted muscle torque was provided to the participants. The discharge patterns of individual motor units (MUs) were studied in three repetitions of isometric plantar flexion at 30 and 60% of Maximum Voluntary Contraction (MVC), before, and 1 day after the 14-day bed rest, respectively. In the GL and GM muscles, the older participants demonstrated higher MU discharge rates than the young, regardless of the contraction level, both before and after the bed rest. In the TA and SO muscles, the differences between the older and young participants were less consistent. Detailed analysis revealed person-specific changes in the MU discharge rates after the bed rest. To quantify the coactivation patterns we calculated the correlation coefficients between the cumulative spike trains of identified MUs from each muscle, and measured the root mean square difference of the correlation coefficients between the trials of the same session (intra-session variability) and between different sessions (inter-session variability) in each participant (intra-person comparison) and across participants (inter-person comparison). In the intra-person comparison, the inter-session variability was higher than the intra-session variability, either before or after the bed rest. At 60% MVC torque, the young demonstrated higher inter-person variability of coactivation than the older participants, but this variability decreased significantly after the bed rest. In older participants, inter-person variability was consistently lower at 60% than at 30% MVC torque. In young participants, inter-person variability became lower at 60% than at 30% MVC torque only after the bed rest. Precaution is required when analyzing the MU discharge and coactivation patterns, as individual persons demonstrate individual adaptations to aging or bed rest.

## Introduction

Several studies demonstrated the decrease of motor unit (MU) discharge rates with age in individual muscles such as first dorsal interosseous (Erim et al., 1999; Vaillancourt et al., 2003), tibialis anterior (TA) (Connelly et al., 1999), soleus (SO) (Kallio et al., 2012), and vastus lateralis (Watanabe et al., 2016), suggesting that age-related loss of muscle strength is related to MU discharge properties. However, a recent study on the TA muscle during isometric contraction at 20% of the maximal voluntary effort (Castronovo et al., 2018) demonstrated that neither the MU discharge rate, nor the coefficient of variation for the interspike interval were associated with age.

One of possible explanations for these discrepancies could be inter-personal differences in muscle coactivation patterns, especially when the investigated movements are controlled by several agonistic and antagonistic muscles. Indeed, the inter-person diversity of muscle coactivation strategies was documented extensively in various conditions (Cutting and Kozlowski, 1977; de Rugy et al., 2012; Horst et al., 2017). In the past few years, person- and condition-specific coactivation patterns of triceps surae muscle were studied extensively (Hug et al., 2019; Aeles et al., 2021), demonstrating that the gastrocnemius medialis (GM), gastrocnemius lateralis (GL), SO and TA muscles exhibit person-specific coactivation patterns, and support person discrimination across measuring sessions. These findings led to the establishment of the concept of person-specific muscle coactivation signatures (Hug et al., 2019).

However, interactions among skeletal muscles are still not understood fully. Most of the previous studies investigated muscle coactivation patterns using amplitude envelopes of the bipolar electromyographic (EMG) signal. This methodology has several limitations. First, it is sensitive to electrode positioning (Vinti et al., 2018; Holobar and Farina, 2021; Vieira and Botter, 2021). Second, amplitude envelopes of EMG reflect not only muscle activation levels, but also the shapes of the motor unit action potentials (MUAPs) and therefore the localization of MU fibers in the MU territory (Vieira et al., 2011, 2016). The uniqueness and inter-person diversity of muscle coactivation strategies as assessed from EMG amplitude envelopes could, therefore, at least partially, originate from person-specific MU fiber localizations in the muscle tissue. Third, EMG amplitude envelopes (in bipolar or multichannel recordings) do not discriminate between the contributions of different MUs, and do not address the separation of contributions from different muscles when significant muscle crosstalk is present (for example, when recording activity of wrist extensors). Fourth, EMG amplitude envelopes are sensitive to changes of MUAPs that are caused either by contraction levels (Vieira et al., 2015), or by muscle shortening and muscle fatigue (Šavc and Holobar, 2021). At least at higher contraction levels, the fatigue may cause significant MUAP changes, decreasing the accuracy of muscle activation estimation (Holobar and Farina, 2021; Šavc and Holobar, 2021). Like MU territories and fibers‘ localizations, the fatiguing profiles and changes of MUAP shapes can be person specific.

Muscle activation patterns were also studied intensively by Non-negative Matrix Factorization (NMF) of the EMG amplitude envelopes (d’Avella et al., 2003, 2015; Tresch et al., 2006). However, it was shown recently that NMF does not remove the MUAP shapes, and is, thus, sensitive to MU fiber localizations, changes of MUAP shapes due to muscle shortening or muscle fatigue (Šavc and Holobar, 2021), and to muscle crosstalk (Šavc et al., 2018). Therefore, also in the case of NMF-based analysis, the inter-person diversity of assessed muscle coactivation patterns could originate from person-specific MU properties.

On the other hand, identification of MU discharges from non-invasively recorded high-density EMG (hdEMG) signals offers a detailed insight into the behavior of relatively large number of MUs (Holobar and Zazula, 2007; Negro et al., 2009; Tanzarella et al., 2021). These EMG decomposition techniques identify the MU discharge times and fully remove the effects of MUAPs from the recorded EMG signals (Holobar and Farina, 2021). They can be adapted to dynamic muscle contractions in which MUAP shapes change significantly (Glaser and Holobar, 2018; Kramberger and Holobar, 2021) and, thus, discriminate between the changes in MUAP shapes and in muscle activation levels. They also offer efficient control of MU identification accuracy (Holobar et al., 2014) and, therefore, control of the quality of the muscle activation estimation. Different approaches to muscle activation estimation from MU discharge patterns were also developed, including Principal Component Analysis of smoothed MU discharge rates (Negro et al., 2009), NMF of smoothed MU discharge rates (Tanzarella et al., 2021) and Cumulative Spike Train (CST) of identified MUs (Farina et al., 2014). Indeed, studies of simultaneous MU behavior in different muscles are increasing (Héroux et al., 2014; de Souza et al., 2018; Kranjec and Holobar, 2019; Davis et al., 2020; Potočnik et al., 2020; Cohen et al., 2021; Tanzarella et al., 2021). However, to the best of our knowledge, comparisons of MU behavior in a group of simultaneously recorded skeletal muscles of older and young persons are still largely lacking.

In this study, we propose a robust approach to estimation of the differences in activation of skeletal muscles. This approach builds on the identification of individual MU discharge patterns and on cross-correlation analysis of CSTs, and is, thus, not sensitive to the person-specific MU fiber localizations. Moreover, it supports quantification of both individual MU properties, as well as interactions between the activation patterns of different skeletal muscles. To demonstrate the efficiency of this approach we examined and mutually compared the person-specific changes of MU behavior in GL, GM, SO, and TA muscles during isometric plantar flexions in older and young participants undergoing a 14-day horizontal bed rest.

Experimental bed rest is a gold standard ground-based model for studying the effects of microgravity, as well as interaction between aging and disuse (Di Girolamo et al., 2021). Post bed rest decreases of muscle mass and strength (Koren, 2015; Pišot et al., 2016; Rejc et al., 2018; Marusic et al., 2021), as well as performance and neural drive (Duchateau, 1995) were reported in the past, along with the damaged neuromuscular junctions and muscle denervation (Monti et al., 2021). Changes in muscle architecture were also reported (Narici and Cerretelli, 1998), although, in a recent study by Koren (2015), the thickness and pennation angle of GM and TA did not change after the bed rest in either young or older persons. However, the studies of muscle activation patterns before and after the bed rest are relatively limited. In some previous bed rest studies (Duchateau, 1995) and immobilization studies (Gondin et al., 2004), amplitude envelopes of bipolar EMG were compared to a supramaximal M wave. Also, Mulder et al. (2009) studied bed rest-induced changes in neural activation properties of the vastus lateralis muscle by using amplitude envelopes of hdEMG. To the best of our knowledge, the individual MU behavior in GL, GM, SO, and TA muscles before and after the bed rest was not studied systematically, even though these muscles were frequently used to study the effect of aging on MU behavior.

In this study, we hypothesize that the previously reported interpersonal variability of muscle coactivations is also demonstrated on the level of MU discharge patterns, and is preserved throughout the aging and bed rest. Therefore, this variability influences the comparison of MU behavior between older and young persons and needs to be considered when analyzing the changes in individual skeletal muscles.

## Materials and Methods

### Experimental Protocol

Fifteen healthy male adults participated in the study: Seven young (aged from 19 to 28 years with mean age of 23.1 ± 2.9 years, height 177.1 ± 5.9 cm, body mass 73.4 ± 7.5 kg) and eight older (aged from 53 to 64 years with mean age of 58.4 ± 3.3 years, height 172.7 ± 4.6 cm, body mass 77.0 ± 11.8 kg). Prior to bed rest all participants underwent medical examination and blood/urine analysis. Exclusion criteria were: Smoking, regular alcohol consumption, ferromagnetic implants, history of deep vein thrombosis, acute or chronic skeletal, neuromuscular, metabolic, and cardiovascular disease conditions, or pulmonary embolism. Participants were well informed of the purpose, procedures and risks involved before signing the informed consent. The study was performed in accordance with the Helsinki Declaration, and approved by the Republic of Slovenia National Medical Ethics Committee (KME 103/04/12).

A 14-day bed rest protocol was conducted in hospital facilities under strict medical supervision, with constant video surveillance and 24-h medical care. A detailed protocol description can be found in a previously published paper (Pišot et al., 2016). The protocol consisted of four main phases:

• 3 days of accommodation to the environment and baseline data collection;
• 14 days of horizontal bed rest with no deviations from the supine position and no active exercises or muscle contraction tests allowed, with a eucalorically controlled diet;
• 2 days of post bed rest ambulatory care;
• 28 days of supervised recovery period (not covered in this study).

### Data Acquisition

Two data acquisition sessions were performed, before (BEFORE), and 1 day after the bed rest (AFTER). Each session consisted of hdEMG and plantar flexion torque measurements during controlled dominant foot isometric plantar flexions with a trapezoidal torque profile. The foot dominance was determined by an experienced physician (as the foot that kicks the ball; all the participants were right-dominant). Each participant’s dominant foot was fixed in a mechanical brace (Wise Technologies, Ljubljana, Slovenia) at 90-degree knee flexion. The exerted muscle torque was measured by an electronic force sensor (HBM, Darmstadt, Germany) at 1,024 Hz. The MVC level was measured after familiarization with the experimental protocol and type of performed contractions. Afterward, each participant performed three repetitions of the plantar flexion at 30 and 60% of MVC torque, with 90-s-long pauses between them. In each repetition the participant performed a 5 s long ramp-up, followed by a 15 s long hold phase and 5 s long ramp-down. The 30 and 60% MVC torque levels were selected to analyze the potential differences between moderate and high contraction levels in both age groups (Watanabe et al., 2016).

During the plantar flexions we recorded the hdEMG of the TA, GL, GM, and SO muscles in the dominant foot. For each muscle we used a flexible grid of 13 × 5 electrodes with 8 mm interelectrode distance (LISiN–OT Bioelettronica, Torino, Italy), resulting in 64 data channels per muscle. The skin was abraded lightly using abrasive paste, and the electrode grids were filled with conductive gel (Meditec–Every, Parma, Italy). A reference electrode was put at the ankle malleolus of the dominant foot. The hdEMG was band-pass filtered (3 dB bandwidth, 10–750 Hz) and recorded in monopolar mode at 2,048 Hz and 12-bit A/D resolution (EMG-USB2 amplifier, OT Bioelettronica, Torino, Italy).

### Data Processing

The torque signal was resampled to the same sampling rate as the hdEMG signals (2,048 Hz), and then both data sets were synchronized with the help of a dedicated trigger signal. The acquired monopolar hdEMG signals were decomposed offline by the Convolution Kernel Compensation (CKC) method (Holobar and Zazula, 2007), yielding binary spike trains of individual MUs. The decomposition results were inspected manually and edited by an experienced human operator, and all the MUs with irregular discharge pattern or with Pulse-to-Noise Ratio (PNR) < 28 dB were discarded (Holobar et al., 2014). This resulted in high accuracy of MU discharge identification.

**FIGURE 1:**
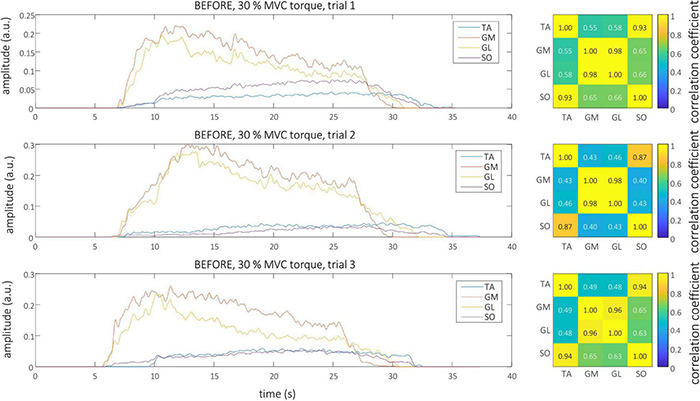
Left, CSTs as calculated from the tibialis anterior (TA), gastrocnemius medialis (GM), gastrocnemius lateralis (GL), and soleus (SO) muscles of older participant (O1) during the three trials at 30% MVC torque before the bed rest. Right, The muscle coactivation as revealed by the cross-correlation analysis of CSTs of different muscles.

**FIGURE 2:**
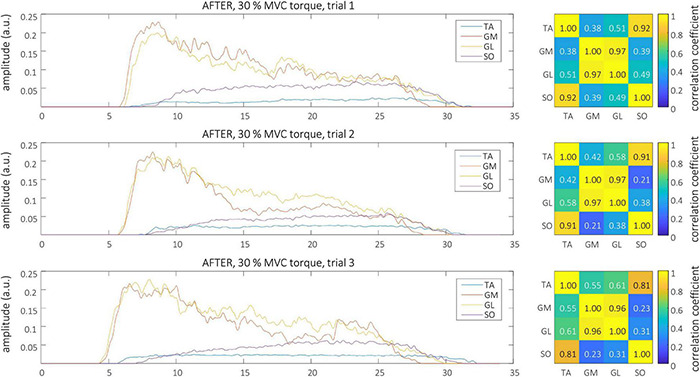
Left, CSTs as calculated from the tibialis anterior (TA), gastrocnemius medialis (GM), gastrocnemius lateralis (GL), and soleus (SO) muscles of older participant (O1) during the three trials at 30% MVC torque after the bed rest. Right, The muscle coactivation as revealed by the cross-correlation analysis of CSTs of different muscles.

Smoothed MU discharge rates (SDRs) were calculated by resampling the instantaneous MU discharge rates to the constant sampling frequency of 100 Hz, and low-pass filtering them by a zero-phase fourth order Butterworth filter with cut-off frequency set to 2 Hz. CST was calculated by summing up the binarized spike trains of individual MUs, and low-pass filtering the result by a 400 ms long Hann window (De Luca, 1985; Negro et al., 2009). Finally, the cross-correlation coefficients between CSTs of simultaneously active TA, GL, GM, and SO muscles were calculated pair-wise to quantify the coactivation patterns of the studied muscles. The Root Mean Square (RMS) difference of CST’s cross-correlation coefficients, calculated between all possible muscle pairs and for all trial combinations, was used to estimate the inter-person, intra-person, inter-session and intra-session variability of muscle coactivation patterns in each individual participant. Autocorrelations of the CSTs were excluded from the RMS difference calculations. Examples of estimated CSTs and their cross-correlation coefficients in a representative older participant are depicted in Figure 1 (BEFORE) and Figure 2 (AFTER).

### Statistical Analysis

Statistical analysis was performed in Matlab version R2021a and JASP software version 0.15. In each sub-analysis we first used the Shapiro-Wilk test to check for normality of the data distribution. For paired comparison we performed the Repeated Measures ANOVA test with Holm-Bonferroni *post hoc* comparisons (in the case of normally distributed data), or Friedman’s Repeated Measures test with Conover’s *post hoc* comparisons and Holm-Bonferroni correction (for non-normally distributed data). The number of factors and their interactions tested are clarified in the Results sections. For unpaired comparison between older and young participants we used the Kruskal-Wallis test whenever the Shapiro-Wilk test indicated that the data were not normally distributed. When normal distribution was not rejected, we used one-way ANOVA. In both cases Bonferroni correction was applied when significant differences were detected.

In all the analyses the Mauchly’s test was used to check the assumption of sphericity, while the Levene’s test was used to check the assumption of homogeneity of variance. When required, we used Greenhouse-Geisser correction for data sphericity. The Omega squared (ω^2^) method was used to estimate the effect size in Repeated Measures ANOVA, with ω^2^ = 0.14 indicating a large effect, ω^2^ = 0.06 indicating a medium effect, and ω^2^ = 0.01 indicating a small effect. For all statistical comparisons the level of significance was set at *p* < 0.05. To evaluate the impact of the limited number of participants included into this study, we used the ANOVA_Power software package (Lakens and Caldwell, 2019) to calculate *post hoc* statistical powers.

## Results

### Number of Identified Motor Units

Because the distribution of the number of identified MUs violated the assumptions of normality, sphericity, and homogeneity, we performed the Friedman’s Repeated Measures test (factors: Session, muscle combination, contraction level). The test didn’t show any differences for the factors session or contraction level. The only difference for the factor muscle was found between TA and GM (*p* = 0.011), with TA having less identified MUs.

To compare young and older participants we performed an unpaired Kruskal-Wallis test. Before the bed rest the number of identified MUs was higher in older than in young participants for the SO muscle at 30% MVC torque, and for the TA and SO muscles at 60% MVC torque. After the bed rest, the number of identified MUs was higher in older than in young participants for the GL muscle at 60% MVC torque, and for the SO muscle at 30% MVC torque (Table 1).

**TABLE 1:** Number of motor units (Mean ± Standard Deviation) identified from different muscles in the BEFORE and AFTER sessions (PNR ≥ 28 dB).

	Older				Young			
	
Session	TA	GM	GL	SO	TA	GM	GL	SO
BEFORE, 30% MVC torque	8.6 ± 4.9	20.5 ± 9.7	14.6 ± 10.4	17.5 ± 5.9*	7.2 ± 4.9	20.5 ± 9.4	10.9 ± 4.9	12.2 ± 5.4*
AFTER, 30% MVC torque	6.3 ± 4.8	17.7 ± 9.8	18.3 ± 7.0	15.7 ± 4.8*	5.5 ± 3.5	17.1 ± 10.1	14.5 ± 7.0	12.3 ± 4.8*
BEFORE, 60% MVC torque	8.2 ± 4.7*	19.3 ± 10.8	11.7 ± 11.2	16.4 ± 8.0*	5.1 ± 3.5*	16.0 ± 15.6	6.4 ± 4.5	8.6 ± 5.5*
AFTER, 60% MVC torque	6.0 ± 3.7	16.6 ± 11.6	12.5 ± 8.0*	13.5 ± 6.9	5.6 ± 3.3	14.2 ± 6.7	7.2 ± 6.2*	12.8 ± 7.9

TA, Tibialis anterior; GM, Gastrocnemius medialis; GL, Gastrocnemius lateralis; SO, Soleus; MVC, Maximum Voluntary Contraction. *Denotes significantly different values in older and young participants (Kruskal-Wallis test, p < 0.05).

### Cross-Correlation of Cumulative Spike Trains

**FIGURE 3:**
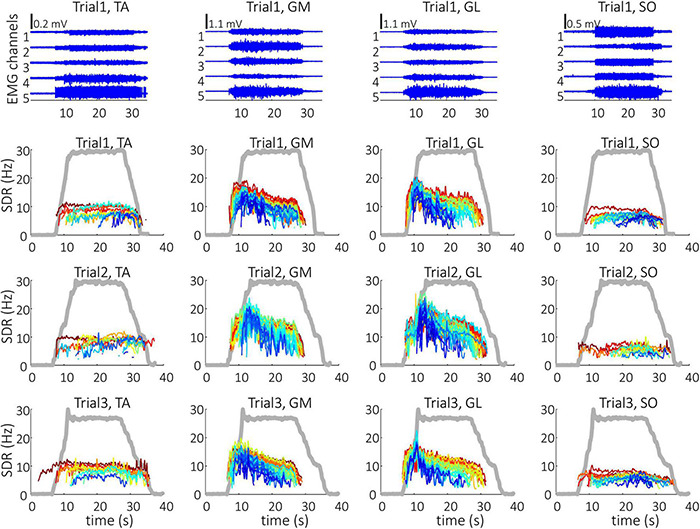
Single differential hdEMG channels (top panels) and smoothed discharge rates (SDR) of individual motor units (panels in the three bottom rows), as identified from the hdEMG signals of older participant (O1). The three trials were performed before the bed rest at 30% MVC torque. TA, Tibialis anterior; GM, Gastrocnemius medialis; GL, Gastrocnemius lateralis; SO, Soleus. The thick gray line represents the measured muscle torque.

**FIGURE 4:**
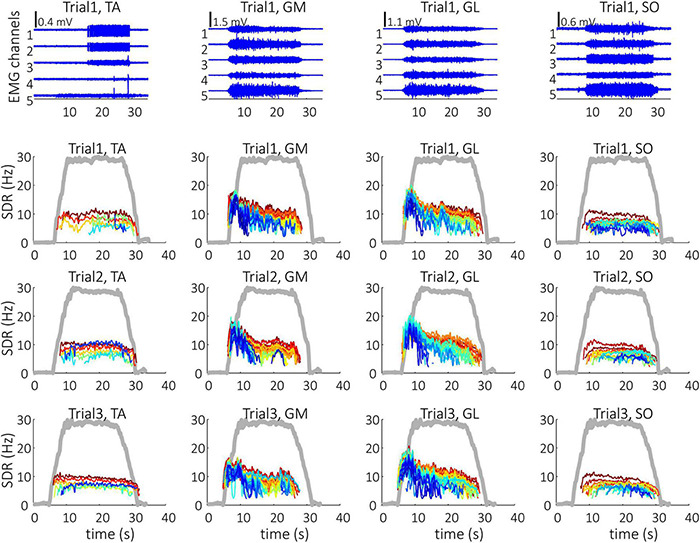
Single differential hdEMG channels (top panels) and smoothed discharge rates (SDR) of individual motor units (panels in the three bottom rows), as identified from the hdEMG signals of older participant (O1). The three trials were performed after the bed rest at 30% MVC torque. TA, Tibialis anterior; GM, Gastrocnemius medialis; GL, Gastrocnemius lateralis; SO, Soleus; The thick gray line represents the measured muscle torque.

**FIGURE 5:**
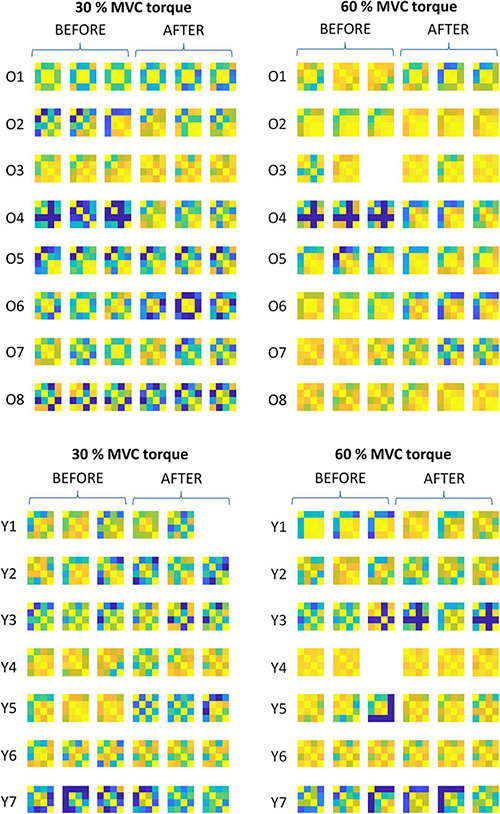
Comparison of the muscle coactivation patterns in older (Oi) and young (Yi) participants across different trials at 30 and 60% MVC torque before (BEFORE) and after (AFTER) the bed rest. The muscle coactivation patterns are coded by pairwise cross-correlation of the cumulative spike trains of different muscles, in the same way as in the right panels of Figures 1, 2. Due to technical problems during the measurements one trial is missing in the O3, Y1, and Y4 participants.

Representative examples of single differential hdEMG signals and the resulting smoothed MU discharge rates at 30% MVC torque at BEFORE and AFTER are depicted in Figures 3, 4, respectively. In each depicted trial and muscle, different MUs are coded with different colors. Note that the identified MUs were activated in different time intervals, demonstrating temporal dynamics of muscle activation. To cope with this dispersion of MU activation intervals, the CSTs were calculated for each muscle, summing up the binary spike trains of all the MUs per muscle. Examples of the estimated CSTs in representative older participant are depicted in Figure 1 (BEFORE) and in Figure 2 (AFTER). The cross-correlation coefficients of CSTs are depicted in the right panels of Figures 1, 2. Note the relatively consistent muscle coactivation pattern in both BEFORE and AFTER sessions in the depicted participant. However, this was not the case for all the tested participants, as depicted in Figure 5. In some participants, the muscle coactivation patterns were relatively preserved across the measuring sessions, whereas, in others, a higher diversity of muscle coactivations was evident, even within the same measuring session. This demonstrates the existence of several degrees of freedom in muscle control strategies during isometric plantar flexion.

In both older and young participants and in both measuring sessions, the cross-correlation of CSTs increased significantly with the contraction level. Since the Shapiro-Wilk test showed that cross-correlation values were not normally distributed, we performed a Friedman’s Repeated Measures comparison (factors: Session, muscle combination, contraction level). This comparison showed that contraction level and muscle combinations have significant effects: For contraction level χ^2^(1) = 20.578, *p* < 0.001; for muscle combinations χ^2^(5) = 55.457, *p* < 0.001. Conover’s *post hoc* comparisons with Holm-Bonferroni correction showed differences between 30 and 60% MVC torque, *T*(321) = 2.123, *p* = 0.034. For muscle combinations all the Holm-Bonferroni corrected *p*-values were not significant, likely also due to the relatively high differences observed in coactivation strategies among the participants (Figure 5). Additional analysis of the simple main effects for the contraction level factor showed higher cross-correlation at 60% than at 30% MVC torque for both BEFORE (*p* = 0.005) and AFTER (*p* = 0.021) in older participants, but only for AFTER in young participants (*p* = 0.003).

### Root Mean Square Differences Between Cross-Correlations

**FIGURE 6:**
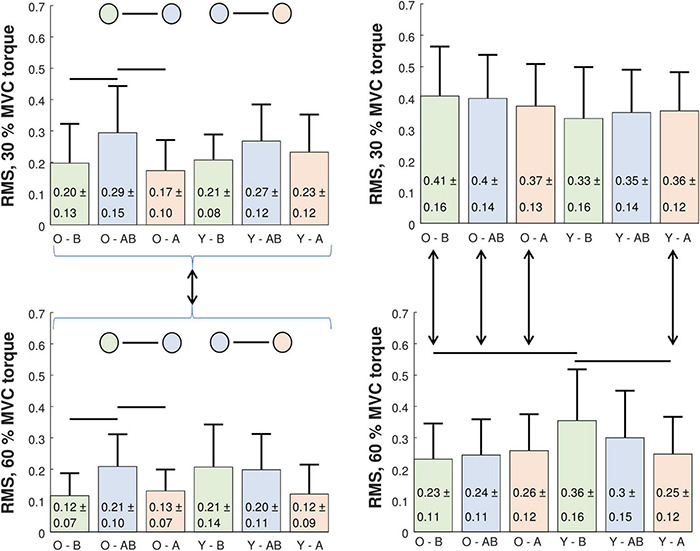
Comparison of intra-person (left panels) and inter-person (right panels) RMS difference of cumulative spike trains‘ cross-correlation coefficients in older (O) and young (Y) participants at 30% (upper panels) and 60% MVC torque (lower panels). RMS differences were calculated across different trials before (B) and after (A) the bed rest (intra-session comparison), and between the trials of before and after bed rest sessions (AB, inter-session comparison). Statistically significant differences were estimated by a three-way Repeated Measures ANOVA test and are denoted by black lines (see the text for a detailed explanation).

Intra-person RMS differences between cross-correlations of CSTs are depicted in Figure 6 (left panels). We calculated the RMS differences for two intra-session comparisons (BEFORE and AFTER) and for one inter-session (BEFORE-AFTER) combination. The RMS differences were normally distributed, and assumptions of homogeneity and sphericity were not violated. Therefore, we performed three-way Repeated Measures ANOVA analysis (between subject factors: Age, within subject factors: Session combination, contraction level). We found large significant effects for the factors session combination [*F*(2, 26) = 10.888, *p* < 0.001, ω^2^ = 0.177] and contraction level [*F*(1, 13) = 8.278, *p* = 0.013, ω^2^ = 0.173], but not for age. All interaction effects were not significant. *Post hoc* comparisons with Holm-Bonferroni corrections revealed higher inter-session RMS differences than intra-session RMS differences (BEFORE-AFTER vs. BEFORE: *p* = 0.014, BEFORE-AFTER vs. AFTER: *p* < 0.001). *Post hoc* comparison for contraction levels showed that the RMS differences were smaller at 60% than at 30% MVC torque (*p* = 0.013). An additional simple main effect test (limited pairwise comparison) for the session combination factor showed that the inter-session RMS differences of older participants were higher than the intra-session at both 30% (*p* = 0.013) and 60% MVC torque (*p* = 0.029), whereas no difference was significant in young. *Post hoc* statistical powers for reported significant RMS differences between CSTs‘ cross-correlation coefficients ranged from 77 to 100%.

Analysis of the inter-person RMS differences between the cross-correlations of CSTs (Figure 6, right panels) followed the same procedure as the analysis of intra-person differences. Since the results were normally distributed, we performed a three-way Repeated Measures ANOVA analysis (between subject factors: Age, within subject factors: Session combination, contraction level). In both inter- and intra-session comparisons we found large significant effects for the main factor contraction level [intra-session: *F*(1, 47) = 47.658, *p* < 0.001, ω^2^ = 0.228; inter-session: *F*(1, 47) = 42.044, *p* < 0.001, ω^2^ = 0.238]. We found medium-sized significant effects for two-way interaction contraction level * age [intra-session: *F*(1, 47) = 12.847, *p* < 0.001, ω^2^ = 0.07; inter-session: *F*(1, 47) = 11.751, *p* = 0.001, ω^2^ = 0.076] and three-way interaction session combination * contraction level * age [intra-session: *F*(1, 47) = 16.242, *p* < 0.001, ω^2^ = 0.07; inter-session: *F*(1.81, 85.062) = 10.568, *p* < 0.001, ω^2^ = 0.053]. Intra-session *post hoc* comparisons revealed differences between the older and young at 60% MVC torque in the BEFORE (*p* = 0.004), but not in the AFTER session (the older had a lower RMS difference). After the bed rest the RMS differences at 60% MVC torque decreased in the young (*p* = 0.005), but not in the older. In addition, the older demonstrated lower RMS differences at 60% MVC than at 30% MVC torque levels for both BEFORE (*p* < 0.001) and AFTER (*p* = 0.002). In young participants the RMS differences were lower at 30% MVC than at 60% MVC torque levels only AFTER (*p* = 0.018), but not BEFORE the bed rest (Figure 6, right panels).

### Smoothed Discharge Rate

Normal distribution of the mean SDR values was not rejected. Therefore, we performed the four-way Repeated Measures ANOVA comparison (between subject factors: Age, within subject factors: Session, contraction level, muscle). We found large significant effects for all three main factors: Session [*F*(1, 12) = 12.460, *p* = 0.004, ω^2^ = 0.131, BEFORE has higher mean SDR], contraction level [*F*(1, 12) = 30.984, *p* < 0.001, ω^2^ = 0.511, mean SDR is higher at 60% MVC] and muscle [*F*(3, 36) = 7.565, *p* < 0.001, ω^2^ = 0.263], but no significant interactions. *Post hoc* comparison between muscles showed that SO had consistently lower mean SDR when compared to TA (*p* = 0.029), GM (*p* < 0.001), or GL (*p* = 0.012). As we were not able to calculate the four-way ANOVA *post hoc* statistical powers in ANOVA_Power software, we calculated three-way ANOVA *post hoc* powers separately for each contraction level. In accordance with the relatively high inter-person differences observed in this study (Figure 5), *post hoc* statistical powers for significant factors in SDRs ranged from 40 to 100% (at 30% MVC torque) and from 23 to 81% (at 60% MVC torque), suggesting that lager cohorts of participants are required to reliably assess the changes of SDRs.

**FIGURE 7:**
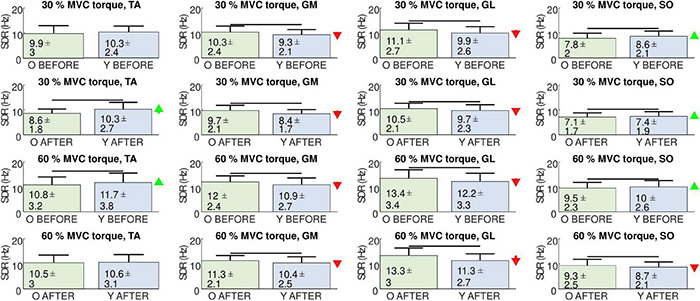
Comparison of smoothed motor unit discharge rates (SDR, Mean ± SD) in the tibialis anterior (TA), gastrocnemius medialis (GM), gastrocnemius lateralis (GL), and soleus (SO) muscles between older (O), and young (Y) participants before (BEFORE), and after (AFTER) the bed rest. The red and green arrows indicate significant decrease and increase of SDR, respectively, at AFTER when compared to BEFORE (one-way ANOVA test, *p* < 0.05).

Figure 7 depicts the comparison between SDRs in individual muscles of the young and older participants. For orientation purposes and comparison with other studies that studied individual muscles, we also show the results of the one-way ANOVA test. In the GM and GL muscles, the MU discharge rates were always higher in the older than in the young participants, regardless of the measuring session or contraction level. The opposite was true for the SO muscle, where the discharge rates were higher in the young than in the older participants, except at the 60% MVC torque in AFTER session. In the TA muscle, young participants demonstrated higher discharge rates than older at 30% MVC torque in AFTER and at 60% MVC torque in BEFORE.

**FIGURE 8:**
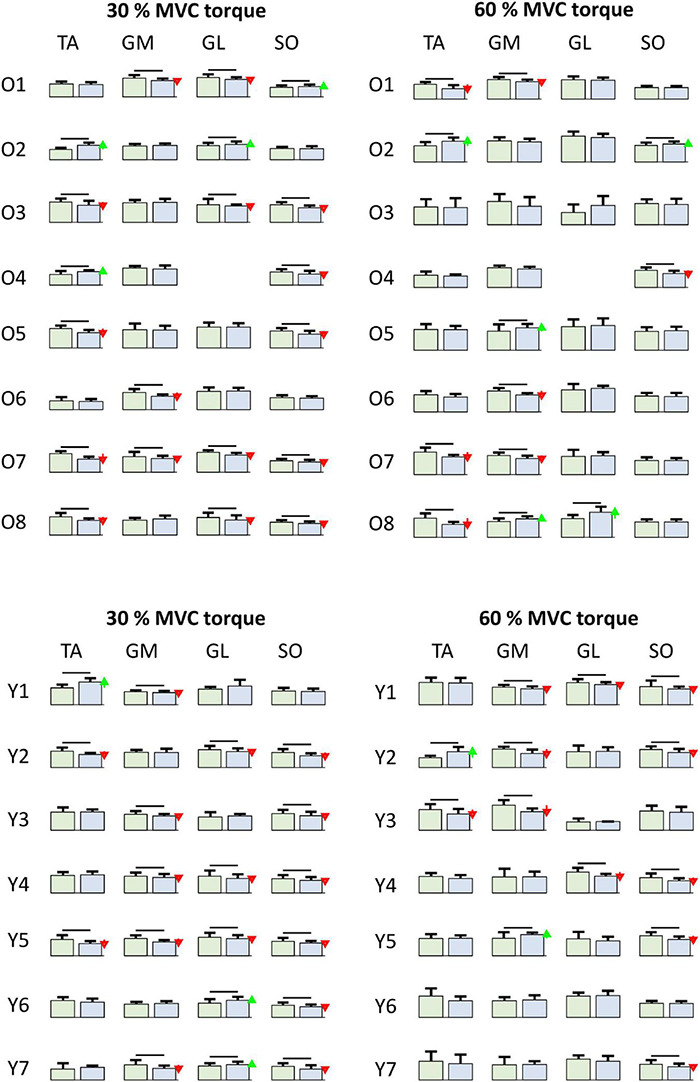
Comparison of smoothed motor unit discharge rates (SDR, Mean ± SD) in individual older (Oi rows, top) and young (Yi rows, bottom) participants for different muscles (columns) at 30 and 60% MVC torque between BEFORE (left green bars) and AFTER (right blue bars). The red and green arrows indicate significant decrease and increase of SDR, respectively, at AFTER when compared to BEFORE (one-way ANOVA test, *p* < 0.05). TA, Tibialis anterior; GM, Gastrocnemius medialis; GL, Gastrocnemius lateralis; SO, Soleus.

Figure 8 depicts adaptations of the SDRs in individual muscles of individual participants. When tested with one-way ANOVA, the SDRs after the bed rest mostly decreased or did not change significantly, though several cases of increases in SDR were also observed. This agrees with the individual changes of coactivation patterns depicted in Figure 5, further demonstrating the person-specific adaptations of load sharing among the GM, GL, and SO muscles after the bed rest.

## Discussion

We identified and compared the discharge patterns in the GM, GL, SO, and TA muscles of eight older and seven young participants before and after 14 days of horizontal bed rest. We demonstrated that the changes of MU discharge rates and muscle coactivation patterns during submaximal isometric plantar flexion after the bed rest are person-specific, and depend on the level of plantar flexion. As discussed in the following paragraphs, our results agree at least partially with many other studies of MU discharge rates in older and young persons (Connelly et al., 1999; Kallio et al., 2012; Castronovo et al., 2018), but also extend them with the analysis across different muscles.

In our study, the number of investigated MUs was relatively high. In total, 3,196, 2,165, 2,453, and 1,179 MU discharge patterns were analyzed in the GM, GL, SO, and TA muscles, respectively. Their quality was assured by selecting the MUs with high PNR (Holobar et al., 2014), and by inspecting the discharge patterns carefully. Noteworthy, the number of identified MUs did not differ significantly across the measurement sessions (before vs. after the bed rest), whereas differences among older and young participants were detected in a limited number of cases (Table 1). This suggests relatively high repeatability of the MU identification in this study.

Although the activation of the TA muscle was not strictly expected, the TA was frequently active during the hold phase of plantar flexion, suggesting that the participants were trying to stabilize the ankle joint. This might be task specific (the participants were instructed to maintain a stable torque during the hold phase). Importantly, since the CKC-based EMG decomposition is biased toward superficial and large MUs (Frančič and Holobar, 2021), we can state with high probability that the activity observed at the skin surface above the TA muscle did not originate from deeper muscles, and was not due to the muscle crosstalk.

The identified MU discharge patterns were used to analyze and compare the MU discharge rates and muscle coactivation patterns mutually. When accumulated across the participants and across the muscles, the MU discharge rates decreased after the bed rest (*p* = 0.004, ω^2^ = 0.131). As expected, the MU discharge rates increased with the contraction levels, and the GL and GM muscles demonstrated higher discharge rates than the SO. In agreement with the diversity of results from other studies that demonstrated decreased MU discharge rates or no significant changes with aging (Connelly et al., 1999; Erim et al., 1999; Vaillancourt et al., 2003; Kallio et al., 2012; Watanabe et al., 2016; Castronovo et al., 2018), the MU discharge rates in our study showed mixed differences between the participants and muscles. When tested with one-way ANOVA, the older participants demonstrated higher MU discharge rates in the GL and GM muscles than the young, regardless of the contraction level or the measuring session (Figure 7). This trend was reversed in the TA muscle, which played the role of antagonist, stabilizing the ankle joint in the studied isometric plantar flexion. At 30% of MVC torque the MU discharge rates in the TA were lower in older than in young participants after the bed rest, but not before. However, at 60% of MVC, the MU discharge rates in the TA were lower in the older than in the young participants before the bed rest, but not after. This agrees with the results of the previous study (Castronovo et al., 2018), where, despite a relatively high number of identified MUs, no association of MU discharge rates with age was observed in the TA muscle during dorsi flexion.

In a previous study by Kallio et al. (2012), the MU discharge rates of the SO muscle were lower in older than in young people, though the differences were not always statistically significant. In our analysis of the SO muscle, the discharge rates between the older and young did not differ extensively, though when tested by one-way ANOVA the differences were significant. During 30% MVC torque, the older participants demonstrated lower discharge rates than the young, both before and after the bed rest. This was also true at 60% MVC torque before the bed rest, whereas after the bed rest the young participants exhibited lower discharge rates than the older ones (Figure 7). This again shows the complexity of interpretation of MU discharge rates on the level of individual muscles, and exposes the need for the person-specific and across muscle assessment of adaptations in muscle control strategies.

Indeed, the detailed analysis revealed individual responses of the participants to the bed rest (Figure 8). In most of the participants, but not all, the MU discharge rates decreased after the bed rest in at least one muscle, but the combination of muscles in which discharge rates decreased was different in different participants. For example, after the bed rest, three older participants decreased MU discharge rates at 30% MVC torque in GM, whereas, in five participants, the differences were not significant (Figure 8). Similar diversity of responses was also evident in the other muscles, for both 30 and 60% MVC torques. Interestingly, in the SO muscle of young participants the discharge rates never increased. Six out of 7 participants at 30% MVC torque, and 5 out of 7 participants at 60% MVC torque had decreased MU discharge rates, whereas in the others, the changes were not significant (Figure 8). These results are at least in partial agreement with the results of the immobilization study on young participants published by Gondin et al. (2004), where decrease of RMS value of bipolar EMG, normalized to respective M-waves, was measured in the SO muscle. The same values were also decreased in the gastrocnemii muscles, although the changes were not significant (Gondin et al., 2004). On the other hand, in the 36 year old participant studied by Duchateau (1995), EMG activity during MVC torque was reduced to a greater extent in the GL (−51%) than in the SO (−32%) muscle, whereas the decrease of the maximal M-wave amplitude was more prominent in the SO (−28%) than in the GL (−12%). Our study confirmed the changes in MU discharge rates, but the responses were heterogeneous and person specific.

To illuminate these differences among different participants better, we calculated the correlation coefficients between the CSTs from GM, GL, SO, and TA before and after the bed rest. In older participants the level of coactivation increased with the MVC torque level in both sessions, before and after the bed rest, whereas in the young this trend was observed only after the bed rest. The differences in muscle coactivation patterns were assessed further by calculating the inter- and intra-person and inter- and intra-session RMS difference of the cross-correlation coefficients of CSTs (Figure 6). In the intra-person comparison, inter-session variability was higher than intra-session variability, both before and after the bed rest. Also noteworthy, the intra-session variability was lower at 60% than at 30% MVC torque level (Figure 6). At 60% MVC torque, the young participants had higher inter-person variability of coactivation than the older ones, but this variability decreased after the bed rest. In the older group, the inter-person variability was consistently lower at 60% than at 30% MVC torque, but in the young participants inter-person variability became lower at 60% than at 30% MVC torque only after the bed rest (Figure 6). This suggests the potential decrease of inter-person differences in plantar flexion control strategies with age.

Our study has several limitations. First, the number of investigated participants that underwent the 14-day bed rest protocol is relatively low, but is comparable to many other studies that compared young vs. older participants (Erim et al., 1999, 20 participants; Connelly et al., 1999, 12 participants; Vaillancourt et al., 2003, 30 participants; Kallio et al., 2012, 17 participants; Castronovo et al., 2018, 20 participants) as well as to other bed rest studies where the number of participants ranged from 6 to 16 (Di Girolamo et al., 2021). This is partially justified by the complexity and stress of the prolonged bed rest that needs to be carried out in a hospital setting with 24-h medical inspection and care. Nevertheless, further investigations on larger cohorts of participants are required to confirm the findings reported herein. Despite this limitation, the number of investigated MUs was relatively large, assuring the high representativeness of the investigated MU discharge rates and CSTs (Farina et al., 2014). Second, to guarantee enough identified MUs, we focused our study on a relatively simple task, comprising isometric plantar flexions with the hold phase targeting only two different contraction levels. More complex tasks would likely expose the differences in cognitive, motor and sensory system interactions of older and young participants, but would also likely increase the heterogeneity of person-specific muscle control strategies, both before and after the bed rest. Also important, MU identification is more prone to methodological errors in dynamic conditions than in isometric conditions. Due to the relatively high complexity of the bed rest protocol, and, consequently, relatively low number of participants, we avoided more complex tasks in our study. Third, we limited our analysis to submaximal voluntary contractions. Increasing the contraction level would likely lead to very high coactivation levels of the investigated muscles. Fourth, to keep the experimental protocol short and to avoid the significant effect of muscle fatigue, we measured only three repetitions of each contraction level. More thorough analysis of repeatability of the observed coactivation patterns across a larger number of experimental recordings is left for future work. Finally, our group of older participants had a mean age of 58.4 ± 3.3 years, whereas the expression “older” is usually used for people of 65 years of age or above. Although this discrepancy might influence the extent of the observed differences, it likely does not change the observation that the adaptations are person specific.

## Conclusion

In conclusion, we demonstrated person-specific changes of MU discharge rates and muscle coactivation patterns during submaximal isometric plantar flexion in both older and young participants after the bed rest. Several differences were observed among the older and young participants. At higher MVC torque levels before the bed rest, young participants demonstrated higher inter-person variability of muscle coactivation patterns than the older, but, after the bed rest, this variability decreased to a level that was comparable to the older participants. Further studies on larger cohorts of participants are required to illuminate the physiological mechanisms behind the observed changes, but our results readily demonstrate that precaution is needed when analyzing the statistical differences in older and young persons, as the inter-person differences of muscle coactivations that are revealed by hdEMG decomposition endure in aging and after immobilization.

## Data Availability Statement

The datasets presented in this article are not readily available because our data was acquired a few years ago, before the GDPR came into force and before open science initiative was developed to the current stage. Unfortunately, this is reflected in our formulation of open data availability statements in the informed consent that the participants signed, which doesn’t mention the possibility of public sharing of the gathered data.

## Ethics Statement

The studies involving human participants were reviewed and approved by the Republic of Slovenia National Medical Ethics Committee. The participants provided their written informed consent to participate in this study.

## Author Contributions

AH, BŠ, MG, and RP contributed to the conception and design of the study and to the data acquisition. MD, AH, GS, and NM decomposed the hdEMG signals. MD, AH, BŠ, and UM performed the analysis of the results and wrote the first draft of the manuscript. All authors contributed to manuscript revision, and read and approved the submitted version.

## Conflict of Interest

The authors declare that the research was conducted in the absence of any commercial or financial relationships that could be construed as a potential conflict of interest.

## Publisher’s Note

All claims expressed in this article are solely those of the authors and do not necessarily represent those of their affiliated organizations, or those of the publisher, the editors and the reviewers. Any product that may be evaluated in this article, or claim that may be made by its manufacturer, is not guaranteed or endorsed by the publisher.

## Abbreviations

CST: Cumulative Spike Train
EMG: Electromyography
GM: Gastrocnemius medialis
GL: Gastrocnemius lateralis
hdEMG: High-density Electromyography
MU: Motor Unit
MUAP: Motor Unit Action Potential
MVC: Maximum Voluntary Contraction
NMF: Non-negative Matrix Factorization
PNR: Pulse-to-Noise Ratio
RMS: Root-Mean-Square difference
SDR: Smoothed motor unit discharge rate
SO: Soleus
TA: Tibialis anterior.
